# Correction to: Local Disordered Region Sampling (LDRS) for ensemble modeling of proteins with experimentally undetermined or low confidence prediction segments

**DOI:** 10.1093/bioinformatics/btaf389

**Published:** 2025-07-17

This is a correction to: Zi Hao Liu, João M C Teixeira, Oufan Zhang, Thomas E Tsangaris, Jie Li, Claudiu C Gradinaru, Teresa Head-Gordon, Julie D Forman-Kay, Local Disordered Region Sampling (LDRS) for ensemble modeling of proteins with experimentally undetermined or low confidence prediction segments, *Bioinformatics*, Volume 39, Issue 12, December 2023, btad739, https://doi.org/10.1093/bioinformatics/btad739

Following publication, the authors identified a bug in the IDPConformerGenerator software that led to some conformations having a mirrored stereochemistry. This does not affect the significance of the reported results but does result in some changes in the main text and the supplement.

The authors have corrected this issue by releasing a new version of the software (January 2024) and associated data files (March 2024), and updating the manuscript accordingly.

The following manuscript corrections have been made:

- To the main body of the text:
  - Added “v0.7.23” in the availability and implementation section of the structured abstract.
  - Changed “v0.7.10" to "v0.7.23” in the first and last sentences on page 2 of the manuscript.
  - Updated Figure 1 with recalculated ensembles with the correct stereochemistry.
  - Changed “Thr35” to “Thr37”, “1828” to “4375”, and “692” to “540” in the Figure 1 legend in accordance with the regenerated ensembles.
- To the supplementary material:
  - Updated Supplemental Figures S2, S3.1, S3.2, S4, S5, S6.1, and S6.2, and Supplemental Table 1.
  - Changed the technical specifications of hardware to the “Niagara” cluster and updating the information with “Each node has two 20 core Intel Skylake CPUs @ 2.4GHz with 202 GB of RAM”.
  - Updated the number of conformers in Supplemental Figure S2, S3.1, S5, and S6.1 legends.
  - Updated the compute cluster used in the legend of Supplemental Table 1.

## Supplementary Material

btaf389_Supplementary_Data

