# Supplementary figures and images for: Local Disordered Region Sampling (LDRS) for ensemble modeling of proteins with experimentally undetermined or low confidence prediction segments
Source: Bioinformatics. 2023 Dec 7;39(12):btad739. doi: 10.1093/bioinformatics/btad739 (PMC10733734; doi:10.1093/bioinformatics/btad739)

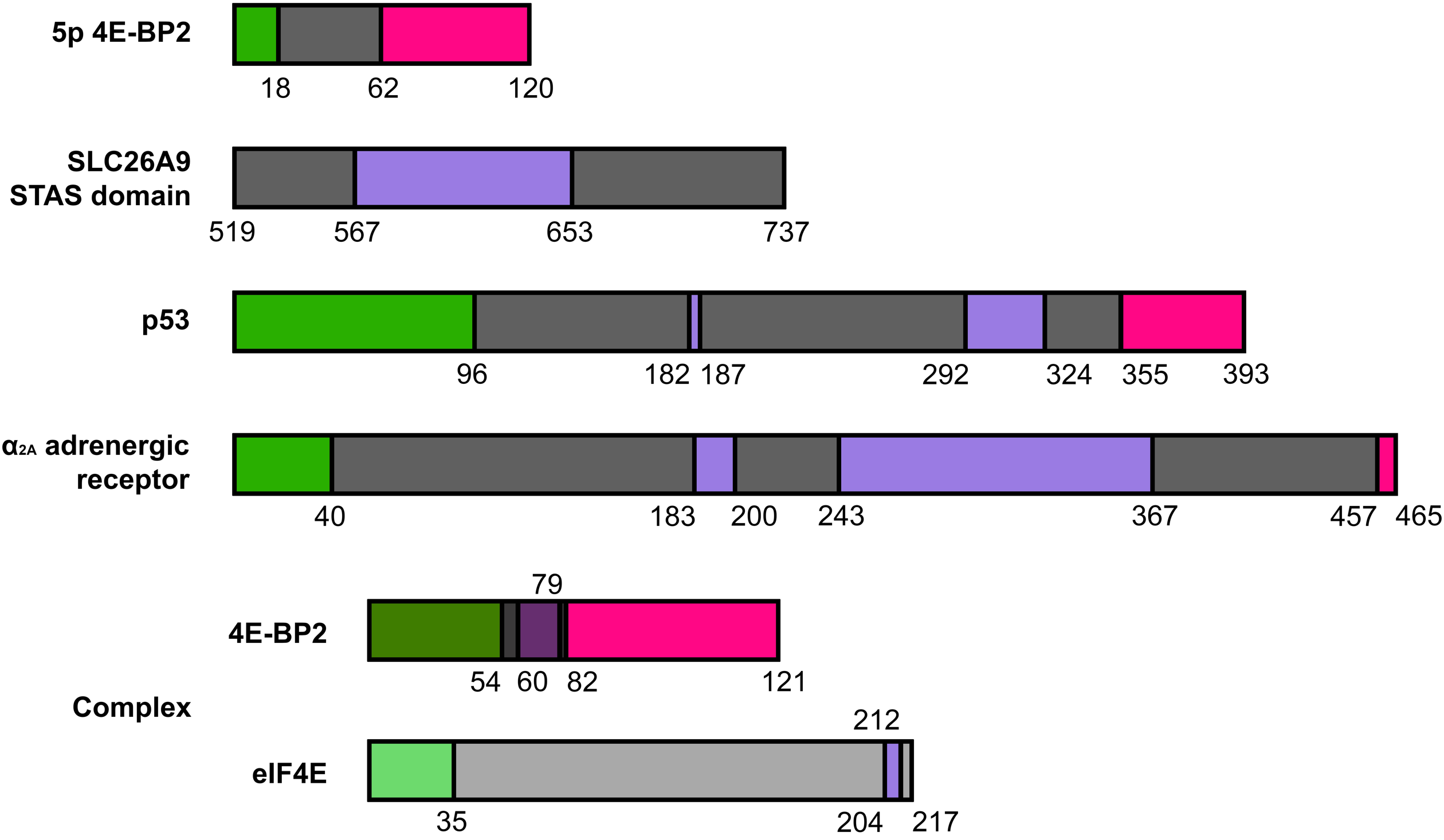

Supplement: btad739_Supplementary_Data [file btad739_supplementary_data.zip › SupplementalFigure1.jpg]

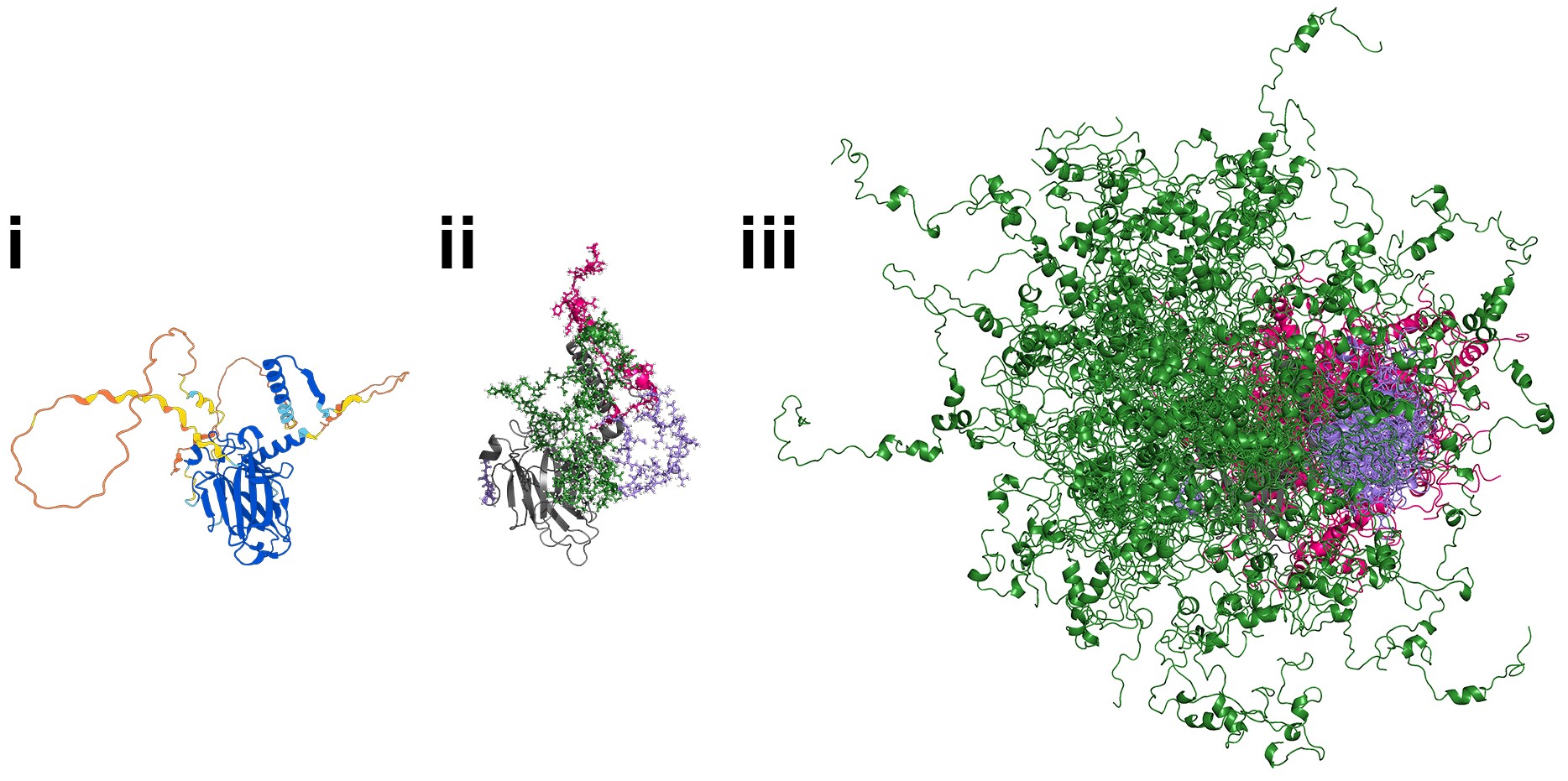

Supplement: btad739_Supplementary_Data [file btad739_supplementary_data.zip › SupplementalFigure2_v2.jpg]

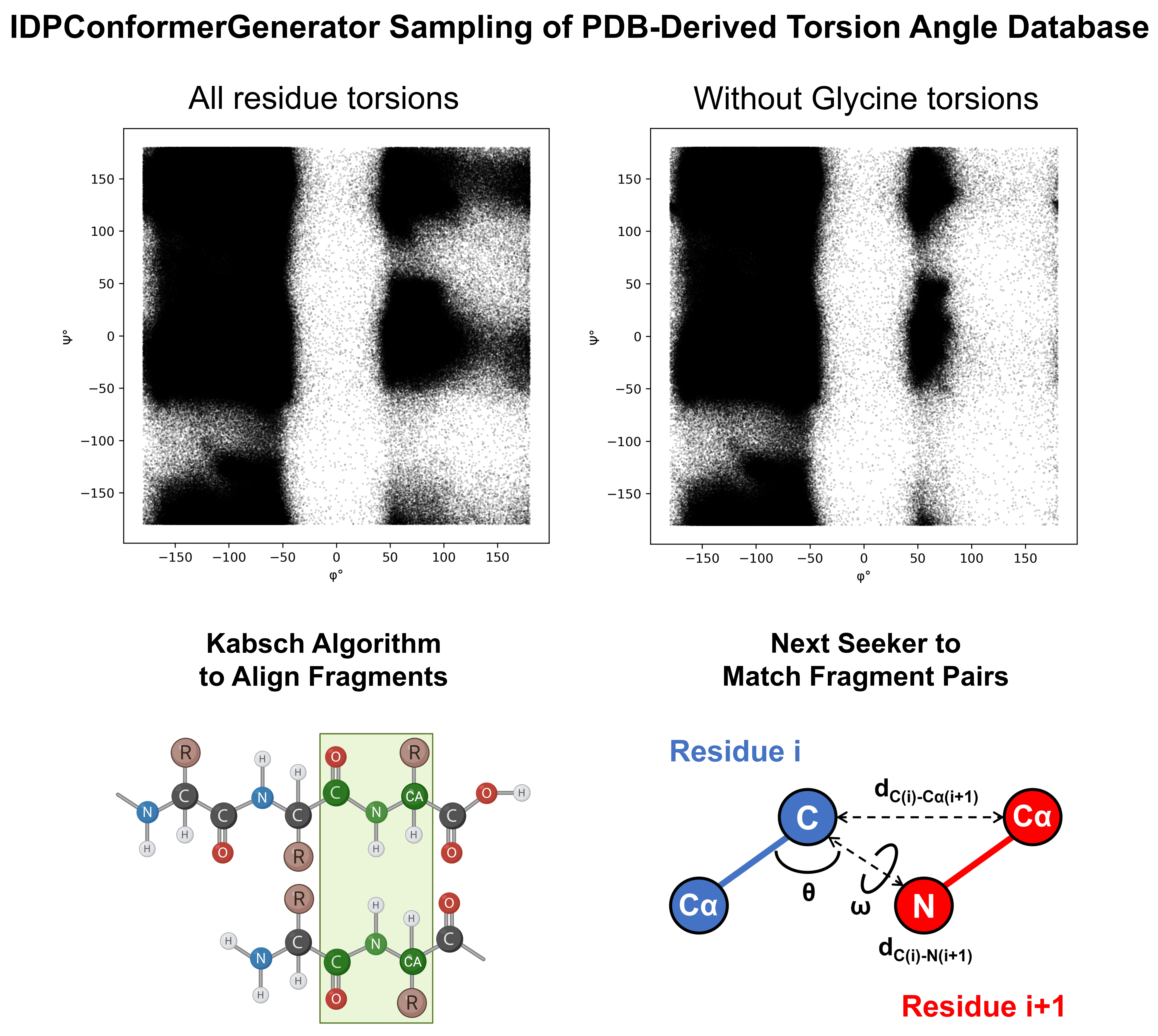

Supplement: btad739_Supplementary_Data [file btad739_supplementary_data.zip › SupplementalFigure7_1_v2_2.jpg]

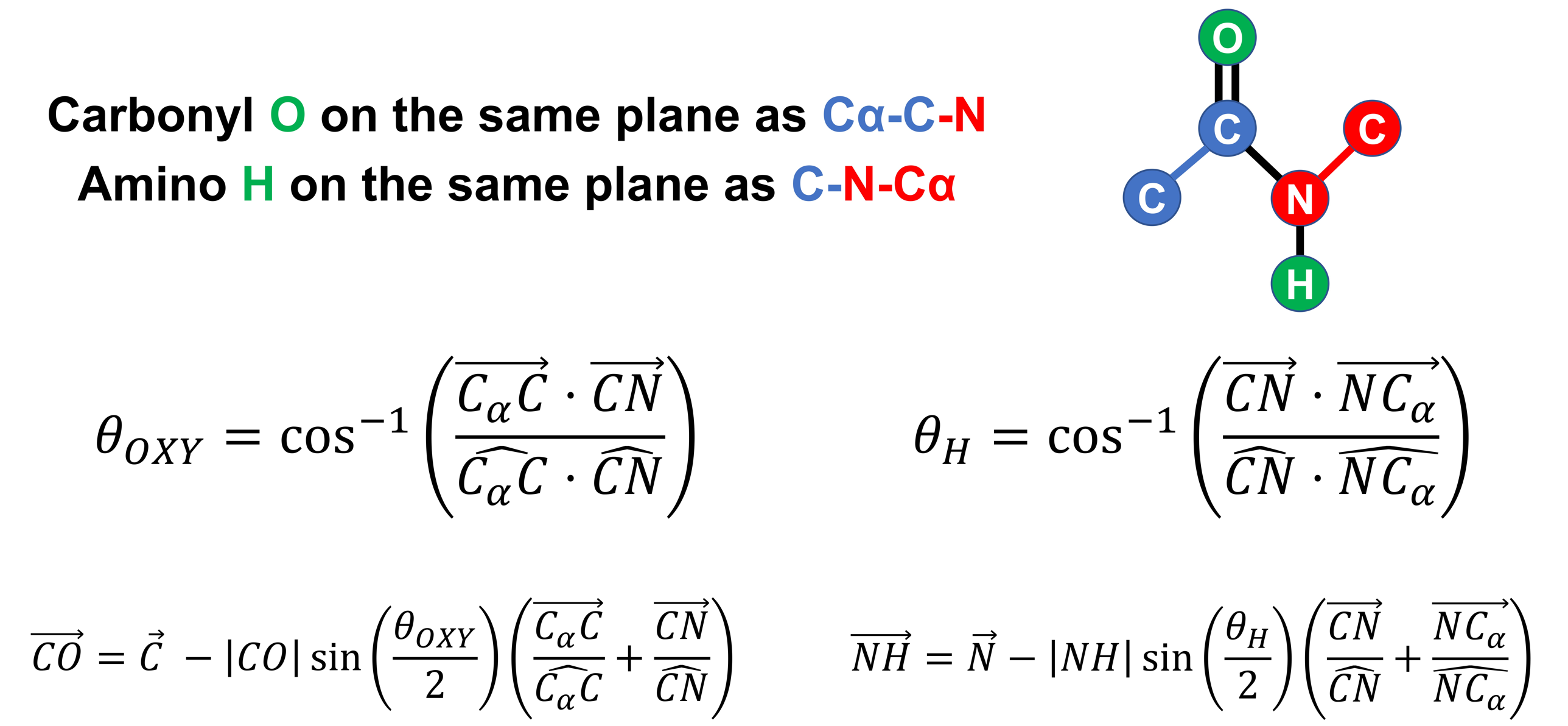

Supplement: btad739_Supplementary_Data [file btad739_supplementary_data.zip › SupplementalFigure7_2.jpg]
